# Methodological Considerations for Cost of Illness Studies of Enteric Fever

**DOI:** 10.1093/cid/ciaa481

**Published:** 2020-07-29

**Authors:** Nelly Mejia, Enusa Ramani, Sarah W Pallas, Dayoung Song, Taiwo Abimbola, Vittal Mogasale

**Affiliations:** 1 Global Immunization Division, Centers for Disease Control and Prevention, Atlanta, Georgia, USA; 2 Policy and Economic Research Department, Public Health, Access and Vaccine Epidemiology Unit, International Vaccine Institute, Seoul, Korea

**Keywords:** typhoid, paratyphoid, cost of illness, methods, economic burden

## Abstract

This article presents a selection of practical issues, questions, and tradeoffs in methodological choices to consider when conducting a cost of illness (COI) study on enteric fever in low- to lower-middle-income countries. The experiences presented are based on 2 large-scale COI studies embedded within the Surveillance for Enteric Fever in Asia Project II (SEAP II), in Bangladesh, Nepal, and Pakistan; and the Severe Typhoid Fever Surveillance in Africa (SETA) Program in Burkina Faso, Ethiopia, Ghana, and Madagascar. Issues presented include study design choices such as controlling for background patient morbidity and healthcare costs, time points for follow-up, data collection methods for sensitive income and spending information, estimating enteric fever–specific health facility cost information, and analytic approaches in combining patient and health facility costs. The article highlights the potential tradeoffs in time, budget, and precision of results to assist those commissioning, conducting, and interpreting enteric fever COI studies.

Estimating the economic burden of enteric fever (typhoid and paratyphoid) is important to evaluate the value and impact of preventive interventions such as vaccination and improvements in access to safe water, sanitation, and hygiene practices. Although ample guidance exists about cost of illness (COI) estimations [1, 2], many of the classic methodologies in this field were developed based on examples from high-income settings with much greater data availability and more sophisticated healthcare pricing, including cost structures and insurance reimbursement schedules, than is typical in settings where enteric fever is prevalent. Estimating COI frequently requires intensive primary data collection efforts in low- and lower-middle-income countries characterized by higher out-of-pocket shares of total health expenditure(s) and only limited high-quality studies published to assess healthcare costs [3–8]. In addition, previous literature has noted the challenge of characterizing the economic burden of enteric fever given the nonspecific nature of its disease symptoms and the tendency toward clinical diagnosis rather than laboratory confirmation by blood culture, particularly in settings with limited laboratory capacity [6, 9].

Accordingly, the aim of this article is to present considerations from 2 large-scale COI studies of enteric fever conducted at sites in 3 Asian and 4 African countries on the methods used as well as the lessons learned from each, and to discuss areas for future research. This article highlights the methodological tradeoffs researchers, evaluators, and program managers are facing when seeking to calculate the economic burden of enteric fever, and implications for interpreting the resulting estimates. However, it does not aim to test the performance of the different methods employed, as the studies were not designed for this purpose. The practical experiences gained from these 2 projects can help inform future efforts to quantify the economic burden of not only enteric fever but also other illnesses in similar settings—such as dengue, cholera, rotavirus, and *Shigella*.

## STUDY SETTING AND STUDY DESIGN COMPARISON

Experiences from the following 2 multicountry enteric fever surveillance projects are presented: the Surveillance for Enteric Fever in Asia Project II (SEAP II), conducted at selected sites in Bangladesh, Nepal, and Pakistan; and the Severe Typhoid Fever Surveillance in Africa (SETA) Program conducted at study sites in Burkina Faso, Ethiopia, Ghana, and Madagascar (the COI component of the program was not performed at SETA sites in the Democratic Republic of Congo and Nigeria).

In both projects, the COI studies were layered onto enteric fever surveillance studies using blood culture for case identification, which identified and enrolled study participants based on well-defined inclusion criteria. In both studies, the study sites were healthcare facilities, not geographically defined areas. In SEAP II, all 7 COI study sites were tertiary or quaternary hospitals in or close to major metropolitan areas, with a mix of public, private nonprofit, and private for-profit facilities. In SETA, 8 study sites were made up of 6 tertiary or quaternary facilities, 1 secondary facility, and 1 primary facility, all of which were public facilities. Differences in epidemiology of enteric fever and other diseases, healthcare settings, healthcare-seeking patterns generally, and service availability by setting make a direct comparison of results difficult, in addition to methodological differences discussed below. The key features of each economic burden study compared to common standard references are summarized in Table 1 [1, 2].

**Table 1.** Comparison of Elements Included in Economic Burden Components Under the Severe Typhoid Fever Surveillance in Africa (SETA) Program Versus the Surveillance for Enteric Fever in Asia Project II (SEAP II)

Study Design Element	Description	SETA	SEAP	Reference Standards [1, 2]
Economic burden components	COI	X	X	X
	Long-term social and economic impact	X		Depends on study objectives
	Quality of life	X		Depends on study objectives
Costs included in COI component	Direct medical costs (eg, diagnosis and treatment), paid by all payers (eg, government, healthcare provider, patient)	X	X	X
	Direct nonmedical costs (eg, transport, food, and lodging, for patient and caregiver), paid by patients and caregiver*s*	X	X	X
	Indirect costs of productivity losses due to morbidity, for patient	X	X	X
	Indirect costs of productivity losses due to patient morbidity, for caregiver	X	X	X
Patient population for patient costs for COI component	Laboratory culture–confirmed cases (enrolled through surveillance component of study)	X	X	Not specified; condition-dependent; inclusion of cases with varying severity and representing different types of health facilities
	Nontraumatic ileal perforation cases (regardless of laboratory culture results)	X	X	
	Clinically diagnosed laboratory-negative cases	X		
	Controls with no febrile illness (matched on age, sex, and geographic residence)	X		
Per-case COI results to be reported	Per laboratory culture–confirmed case, including costs of background health conditions	X	X	Not specified; inclusion of cases with varying severity and representing different types of health facilities
	Per laboratory culture–confirmed case, excluding costs of background health conditions (ie, subtracting healthcare costs of matched controls)	X		
	Per clinically diagnosed laboratory-negative case, including costs of background health conditions	X		
	Per clinically diagnosed laboratory-negative case, excluding costs of background health conditions (ie, subtracting healthcare costs of matched controls)	X		
Method of data collection for patient costs for COI component	Diary card for study participant/caretaker self–record keeping	X		Not specified; condition-dependent
	In-person interview at home or health facility	X		
	Telephone interview		X	
Frequency of data collection from patients for COI component (not including long-term social and economic impact questionnaire for controls in SETA)	In health facility 3–7 d postenrollment/specimen take (1 wk) (all laboratory-confirmed and clinically diagnosed cases)	X		Not specified; condition-dependent
	In household 12–14 d postenrollment/specimen take (2 wk) (all laboratory-confirmed and clinically diagnosed cases)	X		
	In household 28–30 d postenrollment/specimen take (1 mo) (any laboratory-confirmed and clinically diagnosed cases reporting continued illness at previous interview)	X		
	In household 88–90 d postenrollment/specimen take (3 mo) (any laboratory-confirmed and clinically diagnosed cases reporting continued illness at previous interview)	X		
	In household 180 ± 7 d postenrollment/specimen take (6 mo) (any laboratory-confirmed and clinically diagnosed cases reporting continued illness at previous interview)	X		
	In household 270 ± 7 d postenrollment/specimen take (9 mo) (any laboratory-confirmed and clinically diagnosed cases reporting continued illness at previous interview)	X		
	In household 360 ± 7 d postenrollment/specimen take (12 mo) (any laboratory-confirmed and clinically diagnosed cases reporting continued illness at previous interview)	X		
	Telephone immediately after laboratory results (all laboratory-confirmed cases) or discharge (surgical cases)		X	
	Telephone 6 wk (approximately 42 d) postenrollment (all laboratory-confirmed cases) or discharge (surgical cases)		X	
Facility sample for COI component	Facility costs: index sites for surveillance study (including laboratories)	X	X	Not specified; condition-dependent
	Patient costs: any healthcare facility visited prior to patient presentation at index site (assessed through patient recall, not facility-level data collection)	X	X	
Methodology for estimating facility costs	Ingredients-based microcosting	X	X	X
	Activity-based costing	X	X	X
Method of data collection for facility costs	Primary data collection at health facility (observation/interview of key personnel to obtain resource use per procedure)	X	X	X
	Health facility record review	X	X	X
Time frame of COI component (ie, period for which costs are measured)	Facility costs: 12 mo prior to date of data collection	X	X	Not specified; condition-dependent
	Patient costs: duration of episode of illness up to point of interview	X	X	
Analytic horizon of COI component (ie, period for which COI is calculated)	Illness onset through 6 wk (42 d) postenrollment		X	Not specified; condition-dependent
	Illness onset through 90 d postenrollment	X		
	Illness onset through 12 mo postenrollment	X	Planned for cases with complications at 6-wk follow-up, but no such cases identified	
Methodology for valuing productivity losses	Human capital approach	X	X	X
	Friction cost approach	X		X
Perspective of COI component	Societal	X		Societal recommended; depends on study objective
	Healthcare provider (direct medical financial and economic costs)	X	X	
	Patient and caregiver (direct medical financial and economic costs, direct nonmedical financial and economic costs, indirect economic costs)	X	X	
Economic or financial costs included	Economic	X	X	X
	Financial	X	X	X

Abbreviations: COI, cost of illness; SEAP, Surveillance for Enteric Fever in Asia Project; SETA, Severe Typhoid Fever Surveillance in Africa Program.

## SEAP II

The SEAP II project estimated COI for enteric fever from 2 perspectives: (1) patient and caregiver, and (2) healthcare provider. Patient and caregiver COI included self-reported out-of-pocket direct medical costs, for example, for registration, clinical examination, inpatient stay, laboratory tests, medications, and other diagnostic and treatment services; direct nonmedical costs from any funding source (excluding care providers), for example, for transport, food, lodging, and care services for family members; and indirect costs of lost productivity of patients and their caregivers from illness onset until 6 weeks postenrollment (approximately 42 days). Follow-up at 12 months postenrollment of patients reporting continued complications at 6-week follow-up was planned; however, no such cases were identified.

The patients included in the patient and caregiver COI component had to be enrolled in SEAP II surveillance with blood culture–confirmed *Salmonella enterica* serovar Typhi (*S.* Typhi) or Paratyphi (*S.* Paratyphi) or nontraumatic terminal ileal perforation with no known etiology regardless of their blood culture result. From September 2016 to November 2018, 3732 enrolled cases were eligible for participation in the COI study across the 3 countries, of which 3262 patients (or their caregivers) responded to the COI questionnaires (response rate, 87%). COI questionnaires were administered by phone in the local language via trained research assistants to patients at least 16 years of age or their caregivers 2–3 days after laboratory testing or hospital discharge, and again at 6 weeks after enrollment. Patient and caregiver perspective COI per case was calculated as the median of the sum of direct medical and nonmedical costs and indirect costs. The median and interquartile range were reported due to the empirical skewness of the cost data.

Healthcare provider COI included direct medical economic costs (ie, financial outlays plus value of existing and in-kind resources) to the hospitals enrolling patients in the SEAP II surveillance component. Unit costs to diagnose and treat enteric fever cases were estimated through a combination of ingredients-based microcosting for individual procedures specific to enteric fever (eg, blood culture tests, ileal perforation surgery) and activity-based costing for individual procedures that did not differ in resource use between enteric fever and other illnesses (eg, outpatient visit, inpatient bed-day). Data on resource quantities and prices for each procedure including service volumes were collected from each site using annual financial reports, administrative records, on-site observation(s), and staff interviews with clinical, administrative, and financial personnel. Procedure unit costs were multiplied by the frequency of procedures performed for blood culture–confirmed and nontraumatic ileal perforation cases to obtain the average cost per case.

### SETA

The SETA project also estimated COI from the same 2 perspectives as above: (1) patient and caregiver (called “family” COI in SETA); and (2) healthcare provider. Similar to SEAP II, patient and caregiver COI included self-reported out-of-pocket direct medical costs (eg, for registration, clinical examination, inpatient stay, laboratory tests, drugs and medications, and other diagnostic and treatment services); direct nonmedical costs (eg, transport, food, lodging, care services for family members); and indirect costs of lost productivity of patients and their caregivers from illness onset until self-reported recovery. Productivity loss linked to substitute labor was also assessed. These cost components covering the period from fever onset until the patient’s self-reported recovery were collected through a face-to-face interview by trained field surveyors. In addition, diary cards along with instructions for completion were distributed to all patients and caregivers to record their costs between interviews; these cards were referred to by surveyors at each interview time point while eliciting costs as a part of the survey.

The first category of patients eligible for inclusion in SETA comprised blood culture–positive *S.* Typhi cases. As many low-resource settings do not use blood culture for enteric fever confirmation, clinically suspected but blood culture–negative cases (clinical cases) were also included in the SETA COI study and were followed up in the same manner as blood culture–confirmed typhoidal cases. Each clinical case was matched to a blood culture–confirmed *S.* Typhi case by duration of fever, neighborhood, age proximity (± 5 years), and sex—factors that could be potential cost drivers—to better isolate variation in COI attributable to the difference between blood culture–confirmed vs clinically suspected but blood culture–negative cases. As SETA focused on severe typhoid fever, special cases suffering from nontraumatic terminal ileal gastrointestinal perforations (ie, clinically diagnosed typhoid fever gastrointestinal perforation), even in the absence of laboratory confirmation, were included as they are likely to have long recovery time and associated costs [10]. Depending upon self-reported recovery, follow-up was performed up to 7 times starting from 3 to 7 days after enrollment upon earliest availability of blood culture results and continued at 12–14, 28–30, 90 ± 7, 180 ± 7, 270 ± 7, and 360 ± 7 days. The COI interview was discontinued once patients or caregivers reported recovery [11].

The SETA surveillance study design included 4 healthy neighborhood controls (NCs) for each blood culture–confirmed *S.* Typhi case matched by residence, age proximity (± 5 years), and sex. The NCs were asymptomatic at enrollment. An interview of NCs was conducted to collect cost of other illnesses related to any healthcare seeking representing background healthcare costs.

Healthcare provider COI included direct medical economic costs to the hospitalized and nonhospitalized SETA-enrolled blood culture–confirmed *S.* Typhi cases, clinical cases, and special cases. The health facility costs were estimated using an ingredients-based microcosting approach mixed with a macrocosting approach on a standardized tool that collects costs based on the services used. This involved the review of healthcare facility records and the interview of selected clinical, administrative, and financial staff.

The other component of the health economics study under the SETA project is the long-term socioeconomic study (LT-SES). Under this study, the impact of typhoid fever COI on social and financial aspects of patients and caregivers was estimated up to 7 times within 360 days of enrollment matching the COI interview schedule. In addition, a quality of life (QoL) survey tool based on the methods recommend by RAND Health Care [12] was administered at enrollment and at the same interview as LT-SES (7 times in 360 days, up to 8 total interviews for QoL). This allowed tracking the change in QoL from the enrollment up to 360 days.

In addition to typhoid fever, the COI component was also conducted for *S.* Paratyphi and invasive nontyphoidal *Salmonella* (iNTS) disease identified under SETA. This is a single-arm study and does not include special cases, clinical cases, and NCs, nor the LT-SES and QoL study components.

From February 2017 to April 2019, 484 participants were enrolled in the SETA health economics study, of which 98 were blood culture–confirmed typhoid fever cases, 67 were clinical cases, 67 were special cases (suffering from nontraumatic terminal ileal gastrointestinal perforations), 226 were neighborhood controls, 1 was an *S.* Paratyphi case, and 25 were iNTS disease cases.

### Complementary Roles of SEAP II and SETA Economic Burden Components

Both SEAP II and SETA COI studies include elements and methods recommended by standard health economics textbooks (Table 1). These reference texts, however, are not prescriptive regarding many of the practical methodological considerations relating to the estimation of the enteric fever economic burden, which may limit the full comparability of COI estimates across studies. Both studies will produce a COI estimate for comparably defined populations in Asia and Africa from the same perspective over the same analytic horizon, including the same types of costs, namely the economic COI from the patient and caregiver perspective (“family perspective” in SETA). Costs to be measured included direct medical and nonmedical costs, and indirect costs due to productivity losses from illness onset through 12-month postenrollment for blood culture–confirmed cases, without removing background population healthcare costs (ie, without the subtraction of background COI from healthy neighborhood controls, which is done only in SETA). Compared to SEAP, SETA has more frequent time points for patient cost interviews and conducts interviews in-person rather than by phone; SETA also distributes diary cards to study participants to track their time and costs between interviews. In addition, SETA will generate additional estimates of COI for other patient populations (eg, clinically diagnosed cases, special cases, and iNTS disease cases), as well as estimates that control for background morbidity through matching with non–enteric fever controls by geographic location, age, and sex.

The SETA healthcare provider COI uses a mixed method approach in evaluating the average cost expended by the health facilities in treating each illness episode. As health systems and expenditure patterns differ by countries, the SETA and SEAP healthcare provider COI may be mainly applicable to the respective sites, yet can provide a platform for comparison due the large similarity in the approach.

In addition to the COI methods, SETA also includes additional health economics components that are not included in the scope of SEAP. The QoL survey may be able to show the changes in the quality of life over long periods among patients with typhoid fever, which has not been studied before. Similarly, the LT-SES study may demonstrate the social impact of the illness over a longer duration, even postillness. While SEAP II collected information on the socioeconomic status (SES) of enrolled patients, it did not seek to track the effects of enteric fever illness on households’ SES over time or to measure QoL.

## DISCUSSION: METHODOLOGICAL CONSIDERATIONS

Researchers, evaluators, and program managers seeking to understand the economic burden of enteric fever should consider the tradeoffs in the numerous methodological options available for collecting and estimating COI and effects on households’ SES and QoL. These tradeoffs include the following:

### Case Definition/Enrollment Criteria

Given the nonspecific nature of enteric fever symptoms, it has been challenging to estimate the costs due to enteric fever per se, especially in countries in which blood culture testing is not widely available and most cases are clinically diagnosed. The patient inclusion criteria in SEAP and SETA COI studies (Table 1) both included blood culture–confirmed *S.* Typhi or *S.* Paratyphi or nontraumatic ileal perforation cases; SETA additionally included blood culture–confirmed iNTS cases, clinically suspected but blood culture–negative cases, and healthy neighborhood controls. Requiring a positive blood culture test as an inclusion criterion has the advantage of ensuring that the costs collected are for confirmed typhoid and paratyphoid cases. However, this may reduce the sample size of eligible patients in settings without routine blood culture testing or limit patient recruitment to higher-level or private sector health facilities with blood culture testing capabilities, which may introduce selection bias into the patient sample as only certain types of patients are likely to have access to such facilities for reasons of geographic location or price. The net direction of the resulting bias is unclear. Recruiting patients from more expensive higher-level health facilities may result in a higher COI due to higher direct medical and nonmedical costs than for an average case of enteric fever in the country, which would include patients seeking care at less expensive facilities. Such higher-level facilities are often located in urban areas, where indirect costs of productivity losses may also be higher due to higher urban employment and wage rates. However, populations in such settings may have better baseline SES and health, and possibly better access to care that allows more timely diagnosis and treatment, permitting faster recovery (ie, shorter duration of illness) and thus reducing COI. Neither SEAP nor SETA had broad enough facility samples to permit comparison of these potential determinants of COI, nor was it an aim of either study to predict COI based on such factors. Using clinical diagnosis as an inclusion criterion expands the representativeness of the facilities and patients recruited into the study (by reducing selection bias as many health facilities may use clinical criteria for treatment), but risks over- or underestimating COI due to enteric fever alone owing to confounding with other febrile illnesses, with an unclear direction of bias. In other words, inclusion of clinically diagnosed false-positive cases will always overestimate total COI due to enteric fever; on a cost per case basis, however, if clinically diagnosed false-positive cases have higher COI, this would overestimate average enteric fever COI whereas if these false-positive cases have lower COI, this would underestimate average enteric fever COI. To address both concerns of selection bias with only blood culture–confirmed cases and confounding with clinically diagnosed cases, we recommend to report these 2 categories as distinct and independent COI estimates, never merged together. Recruiting patients from a representative sample of health facilities and community sites covering different levels of disease severities is another important consideration to eliminate recruiting biases. Special cases with complications, such as nontraumatic terminal ileal perforation, are recommended for inclusion regardless of the blood culture result, but should be reported separately as they may have unique cost structures and economic burden (eg, higher costs due to surgery). In part, this decision relates to whether the COI study is linked to any other broader surveillance study—as was the case with SEAP and SETA—and what case definitions and recruitment criteria are being used in those studies.

### Controlling for Background Morbidity and Costs

Controlling for background morbidity and costs is a recommended strategy to isolate the COI due to a particular illness, but is not common in the literature on COI in low- and lower-middle-income countries, and is more commonly used in high-income countries with richer data sets [13]. The SETA experience is that identifying sufficiently similar controls for matching with enteric fever cases can be challenging, and that the effort to collect COI from these controls does increase study costs; future SETA results will provide an indication of the extent to which enteric fever COI estimates change after removing background morbidity costs, and will hence inform of the importance of controlling for such costs in future enteric fever COI studies.

### Time Points for Follow-up for Patient COI

Deciding the time points at which to collect patient and caregiver COI involves considerations of frequency, timing, and length of follow-up period. The aim is to minimize recall bias by collecting costs as close as possible in time to when they were incurred (both out-of-pocket expenses and time spent), while minimizing respondent burden, including sensitivity to adding stress to ill patients and concerned caregivers during an episode of acute illness. In SETA, the first COI data collection occurred at the health facility 3–7 days postenrollment upon earliest availability of blood culture results. In SEAP, the first data collection occurred 2–3 days after blood culture confirmation or after discharge in order to use a standard means of data collection by phone after the patient had returned home. In SEAP, all patients were followed up at 6 weeks postenrollment, which was chosen due to budget constraints to match the follow-up time point for the surveillance component as it was hypothesized that most patients’ illnesses would have resolved by 6 weeks. Although further COI data collection was planned at 12 months postenrollment for patients reporting severe complications at the 6-week call, no such cases were identified. In SETA, follow-up COI interviews were conducted at approximately 2 weeks, 1 month, 3 months, 6 months, 9 months, and 1 year postenrollment if the patient reported continued symptoms. Based on 26 months of data collection to date in SETA, 56% and 43% of blood culture–confirmed enteric fever cases and special cases continued to have COI interviews at the 30-day and 90-day time points, respectively. Thus, while both studies planned for up to 1 year of follow-up for severe cases with ongoing complications (which were hypothesized to be the most expensive cases), no such cases were identified after 42 days in SEAP, whereas the SETA COI experience revealed the need for follow-up beyond 90 days (as some residual cost data were observed at the 270- and 360-day follow-up points for some special cases in SETA). Where budget permits, more frequent data collection between diagnosis and final follow-up is preferred to reduce recall bias, given that many cases of enteric fever resolve relatively quickly (range of length of illness, 13–21 days) [3, 6, 7]; for those with ongoing complications, follow-up for up to 1 year is recommended where research budget allows.

### Modality of Data Collection

**Figure 1.**
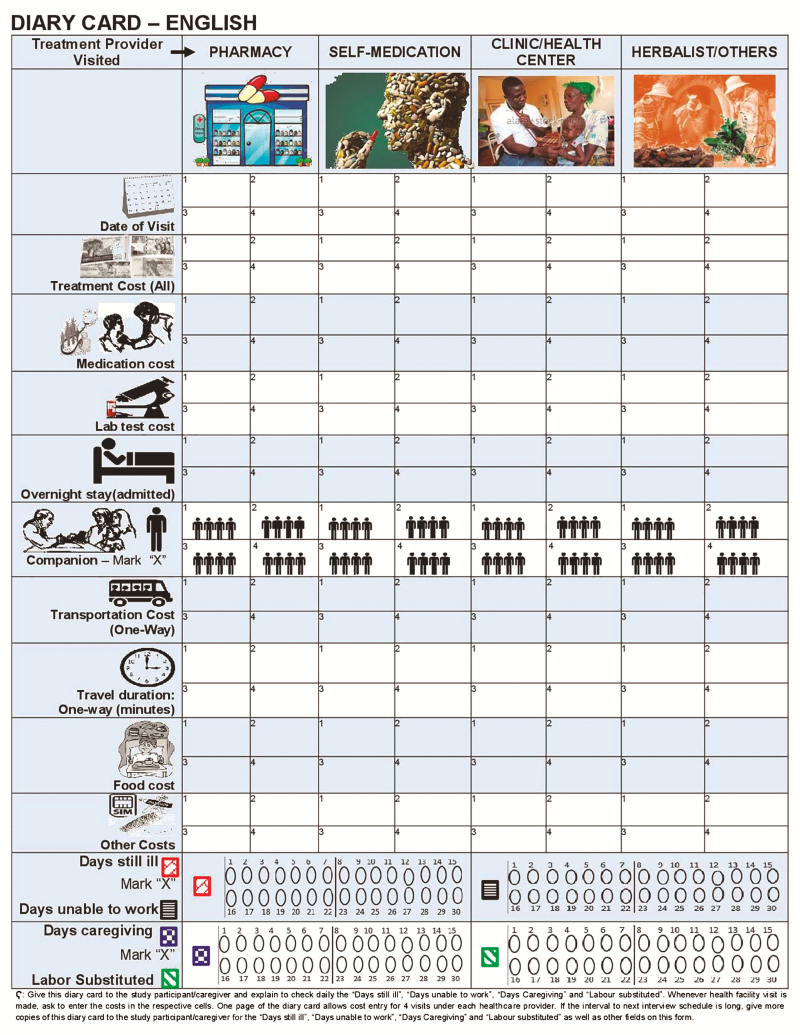
Example of Severe Typhoid Fever Surveillance in Africa (SETA) project diary card for patient and caregiver tracking of out-of-pocket expenses and time use related to episode of enteric fever.

SETA collected data in person at health facilities and patients’ homes, whereas SEAP II collected information over the phone due to budgetary constraints. If the budget allows, in-person data collection permits potentially greater rapport building with the respondent, better understanding of the questions and responses, and data collector observation of contextual information that may help validate certain responses (eg, of SES). The effect of data collection modality on the response rate is unclear; both SEAP and SETA experienced challenges in contacting participants to arrange interviews, and it is not known to what extent the prospect of a home visit from a data collector may have been a deterrent or an incentive. In some SETA sites, participants were reimbursed for travel expenses to come to the health facility for a follow-up COI interview rather than the data collector traveling to their homes; it is not clear if this may have been an incentive to participate or may have been preferred to a home visit (eg, provided more anonymity to the respondent). SETA also distributed printed diary cards to patients and caregivers to track their expenses and time spent related to the illness between interviews (Figure 1). However, not all patients and caregivers completed these diary cards, so their value to improve precision or save time for data collectors during follow-up interviews is unclear. Both studies collected potentially sensitive information on out-of-pocket expenses, wages, household assets, and other socioeconomic characteristics. Such information may be more easily collected in person, although this may depend on cultural, political, and economic factors. For example, many SEAP II study participants refused to provide income information, especially in settings in which concerns were expressed that the information would/could be reported to government authorities for taxation or other purposes (or even that the phone calls for data collection were being monitored by government for such purposes, even if the study team did not intend this).

### Methods for Valuing Productivity Loss

Standard methods for valuing productivity losses include the human capital approach and friction cost approach [1, 2, 14]. In low- and lower-middle-income country settings in which underemployment is prevalent and with large informal sectors and unpaid home production labor, it is typically assumed that replacing labor is “frictionless” and thus a human capital approach is more appropriate, with the challenge being to assign relevant wage rates [3, 6, 7, 15]. SEAP used the human capital approach and asked for monthly income equivalents in wage bands so that the respondent did not have to provide an exact amount but merely the band; the median of each band was then applied to value the respondent’s time. A conservative approach to estimating productivity losses was taken to value only the time of those who lost income or used paid sick leave days. The time of caregivers who reported not earning income but who took time away from their normal activities to accompany a patient to seek and receive care was valued at the median wage reported across wage-earning caregivers in the sample, with sensitivity analyses conducted at each country’s minimum wage rates. By comparison, in addition to the human capital approach, SETA asked a subset of questions to caregivers to try to measure friction costs. The SETA COI inquired whether the caregivers or patients (if adults) had unfulfilled tasks during the duration of their illness and whether they could fulfill it by working extra after recovery. Neither SEAP nor SETA values future productivity losses due to death and long-term disability of patients. Both studies only calculate the productivity losses of patients and caregivers up to the date of the last questionnaire answered within the 12-month analytic horizon of each study.

### Representativeness of Health Facilities at Which Patients Are Recruited and Costs Collected

In the SEAP study, patients were only enrolled via health facilities that had capabilities for blood culture testing, which limits the patient sample to those with access to these higher-level hospitals (mostly private) in urban areas. Due to budget constraints, healthcare provider COI was also conducted in these same enrollment sites. As a result, the COI estimates for SEAP II are not nationally representative and cannot be generalized to estimate the economic burden of enteric fever in the study countries. The SETA COI study also enrolled patients through the public health facilities selected for the surveillance component. These facilities are either already equipped with blood culture diagnostic systems or those systems were set up by SETA to ensure that laboratory testing standards were the same across all study countries. Considering large geographical variations within SETA countries, caution is advised in generalizing the findings from these sites. Future research should consider the intended aim of the economic burden estimation for decision making when selecting the patient and facility sample, and aim for representative samples when extrapolation to the national level is desired.

### Combining COI From Multiple Perspectives

Neither SEAP II nor SETA has the explicit ability to capture COI from the true societal perspective of the study countries, which would include costs to all payers including government; private sector; insurers; healthcare providers; patients, caregivers, and their families; and residents in the country in general. In SEAP II, the prices that patients paid for procedures at health facilities were different than those procedures’ economic costs (ie, value of resources used) from the healthcare provider perspective. For some procedures, patients paid more than the cost to the hospital to conduct the procedure, whereas for others the hospital was charging less than it cost the hospital to conduct the procedure. One approach to combining patient and caregiver direct medical costs with those from the study site hospitals would have been to simply use whichever estimate was larger (patient or healthcare provider) to reflect the opportunity cost of resources to the society (eg, if a patient paid more than it cost the hospital to conduct the procedure, these patient resources above the production cost could have been applied to alternative uses). In the SEAP study, however, the costs were not combined across perspectives because of the difficulty in “netting out” the producer and consumer surpluses lost, and because of limited information on healthcare provider COI from lower-level sites beyond the surveillance study hospitals at which patients reported receiving care.

SETA study sites are located in public facilities; therefore, their healthcare provider costs are part of COI from the government perspective. As in SEAP, however, the healthcare provider perspective COI cannot be combined together directly with the patient and caregiver (family) perspective COI to estimate societal costs, because there is overlap as some of the out-of-pocket expenses borne by patients and caregivers were paid to government facilities. As the study captures only average health facility costs unlinked to individual enteric fever cases and the out-of-pocket expenses collected at the individual level, the deduction of this overlapping cost component has operational challenges. Thus, the direct SETA study output will be one COI from the government perspective (for these public-sector healthcare provider costs) and another from the patient/caregiver (family) perspective. SETA will deploy statistical methods such as the deduction of average out-of-pocket payments made by the patients and caregivers to the health facility to avoid double counting, and then combine costs borne by government and the patient/caregiver to provide a partial estimate of the societal costs.

## CONCLUSIONS

Understanding the economic burden of enteric fever in specific low-resource contexts provides critical information to decision makers who must allocate scarce resources across prevention and treatment for this and other health conditions. SEAP II and SETA are providing up-to-date estimates of the economic burden using robust standardized methods across large multicountry samples; these results will help inform considerations of introducing the new typhoid conjugate vaccine and other interventions related to water, sanitation, and hygiene to prevent and control enteric fever. This article has summarized the approaches taken by both studies toward estimating the economic burden of enteric fever, enhancing transparency and aiding in the interpretation and comparison of results across studies. Theoretically, there are advantages to the more intensive and expansive data collection conducted under SETA; however, this requires a larger study budget, and the value that this additional data will provide for decision making (eg, how high the background healthcare costs are in the population and, hence, whether subtracting these background costs via matched neighborhood controls substantially reduces the economic burden attributable to enteric fever) is not yet clear. We recommend the reference standard methods (as seen in Table 1) as applicable to research questions and affordable given study budgets; if budget permits, we recommend that COI from a societal perspective, in-person interviews for patient costs, and inclusion of enteric fever cases with varying severity, recruited from a representative sample of health facilities and communities, should be pursued. Inclusion of analyses of changes in SES and QoL depend on study objectives.

Although it is not possible to formally evaluate the relative precision of the different methods used, listing the advantages and disadvantages of the various options offers practical guidance to researchers and funders in designing future studies. Future research may wish to consider testing several of these options to determine impact of different methodological choices on precision of estimates; the practical relevance of investing in such methodological research could be explored through modeling of the value of information. Future research should also consider the separate but related question of determinants and correlates of enteric fever COI, such as disease severity, geographic residence, patient age, patient sex, and patient SES; COI studies should collect data on these variables to permit disaggregation of COI by these dimensions and exploration of drivers in COI variation. Health economics researchers, decision makers, and funders should consider what degree of precision is needed in economic burden estimates to help determine the appropriate methods and research budgets required to conduct future economic burden studies that will meet decision makers’ needs.

## Notes

***Acknowledgments.*** The authors acknowledge the contributions made by the Surveillance for Enteric Fever in Asia Project II (SEAP II) and Severe Typhoid Fever Surveillance in Africa (SETA) Program study site teams to cost of illness data collection.

***Disclaimer.*** The views are those of the authors and do not reflect the official position of the US Centers for Disease Control and Prevention (CDC) or the International Vaccine Institute (IVI).

***Financial support.*** This work was supported by the Bill & Melinda Gates Foundation (BMGF), and the CDC. The research by the IVI was funded by the BMGF (grant number OPP1127988). The IVI acknowledges its donors, including the Republic of Korea and the Swedish International Development Cooperation Agency.

***Supplement sponsorship.*** This supplement is funded with support from the Coalition against Typhoid Secretariat, housed at the Sabin Vaccine Institute in Washington, DC and made possible by a grant from the Bill & Melinda Gates Foundation.

***Potential conflicts of interest.*** The authors: No reported conflicts of interest. All authors have submitted the ICMJE Form for Disclosure of Potential Conflicts of Interest.
